# The Graduating European Dentist Curriculum Framework: A Multi‐Stakeholder View

**DOI:** 10.1111/eje.70028

**Published:** 2025-08-14

**Authors:** James Field, Sibylle Vital, Denis Murphy, Jonathan Dixon, Julia Davies, Argyro Kavadella, Mohammed Al‐Haroni, Paul Brady, Blanaid Daly, Michael Dolan, Filip Galo, Cedric Grolleau, Dympnah Kavanagh, Albert Leung, Paul Lyons, Maria‐Cristina Manzanares, Upen Patel, Gitana Rederiene, Miguel Pavão, Maria João Ponces, Barry Quinn, Nibal Sabri, Ross Scales, Ina Schüler, Saulė Skinkytė, Stephanie Tubert‐Jeannin, Katleen Vandamme, Brian O'Connell

**Affiliations:** ^1^ Association for Dental Education in Europe (ADEE) Cardiff University Cardiff UK; ^2^ ADEE University Paris Cite Paris France; ^3^ ADEE Trinity College Dublin Dublin Ireland; ^4^ ADEE The University of Sheffield Sheffield UK; ^5^ ADEE University of Malmo Malmo Sweden; ^6^ ADEE European University Nicosia Cyprus; ^7^ ADEE The Arctic University of Norway Tromsø Norway; ^8^ Cork University Cork Ireland; ^9^ Trinity College Dublin Dublin Ireland; ^10^ Department of Health Dublin Ireland; ^11^ EDSA (European Dental Students Association) Dublin Ireland; ^12^ FEDCAR (Federation of European Dental Competent Authorities and Regulators) Paris France; ^13^ CECDO (Council of European Chief Dental Officers) Dublin Ireland; ^14^ Royal College of Surgeons Dublin Ireland; ^15^ Irish Dental Council Dublin Ireland; ^16^ ADEE University of Barcelona Barcelona Spain; ^17^ ADEE University of Birmingham Birmingham UK; ^18^ EDHF (European Dental Hygienists Federation) Utrecht the Netherlands; ^19^ Portuguese Dental Association Porto Portugal; ^20^ ADEE University of Liverpool Liverpool UK; ^21^ ADEE Dublin Ireland; ^22^ General Dental Council London UK; ^23^ ADEE University of Jena Thuringia Germany; ^24^ EADPH (European Association for Dental Public Health) Marburg Germany; ^25^ ADEE Université Catholique de Louvain Ottignies‐Louvain‐la‐Neuve Belgium

**Keywords:** consensus, dentist, education, oral health professional, review

## Abstract

In 2025 the Association for Dental Education in Europe (ADEE) Graduating European Dentist (GED) taskforce held an international multi‐stakeholder event that undertook a deep‐dive into the perceived ideologies underpinning Oral Health Professional (OHP) education. This paper reports how the event was planned and conducted—and reports the challenges that were discussed in relation to delivering OHP education, potential solutions to each challenge, and priorities for which the ADEE GED taskforce should focus its activity. Due to the very collaborative and fruitful nature of this event, ADEE plans to hold further multi‐stakeholder meetings across Europe.

## 1 Introduction

Over 25 years ago, the original EU‐funded Thematic Network Project (DentEd) aimed to facilitate the convergence of Dental Education across Europe. DentEd's three interlinked projects considered the profile and competences of a graduating European dentist, the curriculum and methods of quality assurance. All three have proved instrumental in shaping the delivery of dental education across Europe since that time [1, 2, 3]. In 2015, a new taskforce was established to revisit, reconsider and accordingly revise the content and the ideologies that should underpin a modern European dental curriculum. At the time, the taskforce used a curriculum ideology inventory approach to help shape their work—considering Schiro's 4 main ideologies (Scholar Academic, Learner‐Centred, Socially Efficient and Socially Reconstructive) [4]. The taskforce, which included student representation from the European Dental Students Association (EDSA), concluded that not one single curriculum ideology satisfied the needs of the Graduating European Dentist (Table 1). The result was a newly configured suite of learning outcomes that provided a basis from which graduates could build confidence and competence towards becoming independent practitioners who would accept the importance of continuing professional development throughout their career.

**TABLE 1:** Suitable curriculum ideologies (Schiro) that were considered by the 2015 taskforce.

Curriculum element	Ideology	
Purpose	Learner centred	Socially reconstructive
Teaching	Learner centred	
Learning	Socially efficient	
Content	Scholar academic	
Student outcomes	Socially efficient	
Evaluation	Socially efficient	

**FIGURE 1:**
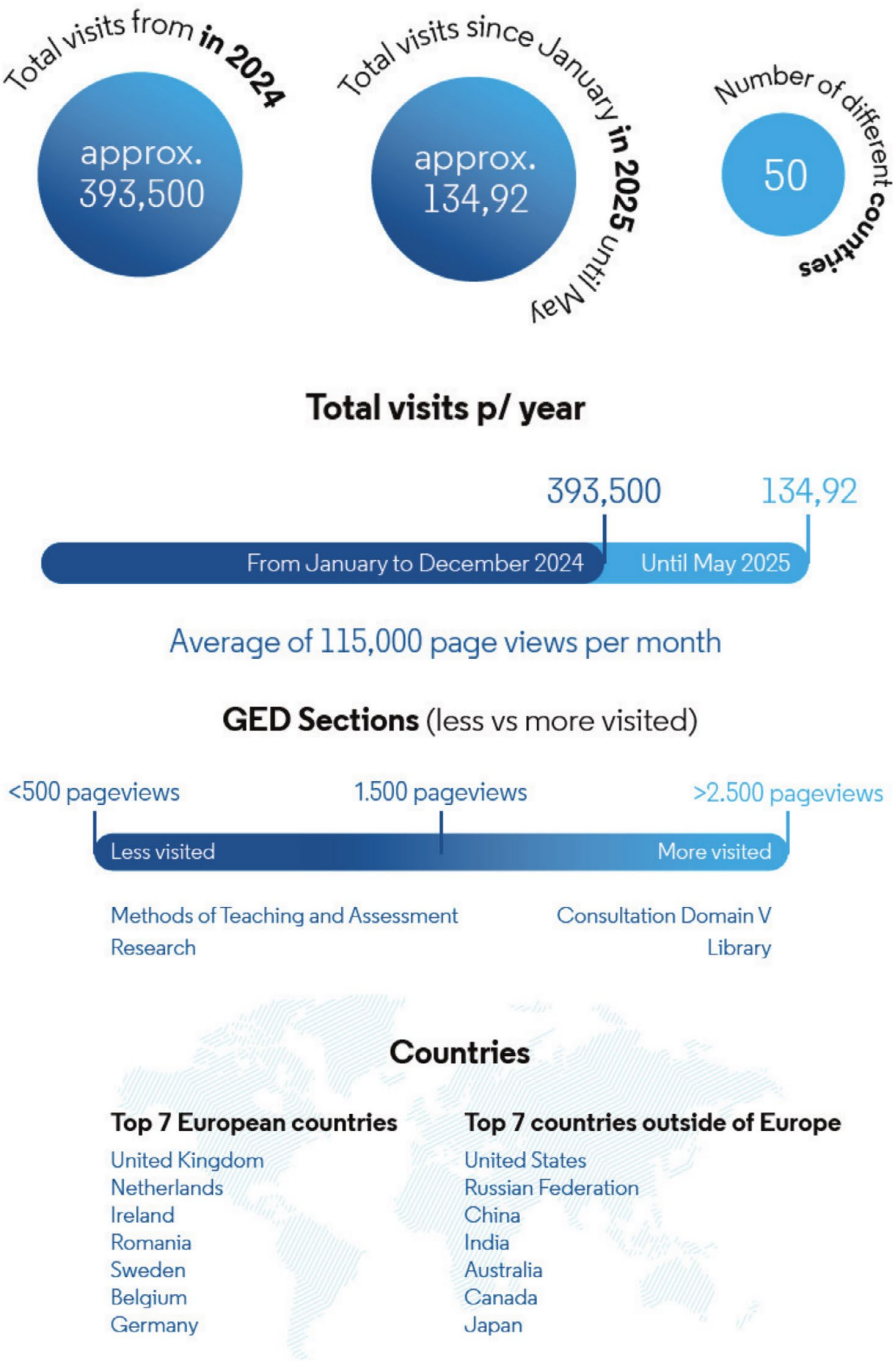
Usage statistics for the GED website.

Since its publication in 2017, the Graduating European Dentist (GED) curriculum framework has proven to be very popular with educators, as demonstrated by the fact that the documents themselves have been cited almost 500 times, emerging as a key reference for discussing the expectations of graduate dentists across Europe. Further, the GED web resource (https://adee.org/graduating‐european‐dentist) which provides access to the most recent version of the curriculum, a supplementary curriculum library and other interactive features, has been viewed over 1.3 million times, averaging nearly 115 000 page views per month—and visited by over half a million unique visitors over the past 2 years (Figure 1). Table 2 outlines the citation statistics for the GED suite of papers—although now that the GED continually evolves online, the most recent review paper from the GED taskforce team should be used going forward, in order to reference the GED curriculum [5].

**TABLE 2:** Papers, links and citations for the GED curriculum.

Paper title	DOI	Authorship	Citations at time of print
7‐year review Commentary	https://doi.org/10.1111/eje.13058	Field et al. 2025 [5]	4
Original Commentary and Introductory paper	https://doi.org/10.1111/eje.12307	Field et al. 2017 [6]	222
1: Professionalism	https://doi.org/10.1111/eje.12308	McLoughlin et al. 2017 [7]	42
II: Safe and Effective Clinical Practice	https://doi.org/10.1111/eje.12309	Field et al. 2017 [8]	40
III: Patient‐Centred Care	https://doi.org/10.1111/eje.12310	Field et al. 2017 [9]	52
IV: Dentistry in Society	https://doi.org/10.1111/eje.12311	Gallagher et al. 2017 [10]	30
V: Research	https://doi.org/10.1111/eje.13040	Field et al. 2024 [11]	2
Methods of teaching and assessment	https://doi.org/10.1111/eje.12312	Field et al. 2017 [12]	84

*Note:* Citation information provided by Google Scholar.

Data collection across Europe through the European Union (EU)‐funded Erasmus+‐funded project ‘O‐Health‐Edu’ has shown that the GED is utilised locally by almost 60% of responding schools [13]. This demonstrates the positive impact that GED is having on a local level with individual institutions. In November 2023, the Federation of European Dental Competent Authorities and Regulators (FEDCAR) endorsed the use of the GED curriculum framework—and even prior to this, the framework had already been supported by some national regulatory bodies such as the UK's General Dental Council; further, the Irish Dental Council had also already adopted the GED framework as a basis for their national curriculum.

Despite the progress marked by the 2017 GED framework, it has become increasingly evident that the educational and political landscapes across Europe have evolved substantially over the past decade. Longstanding recommendations—such as promoting early clinical exposure, embedding contextually relevant content in relation to the medical sciences, and incorporating leadership and management training—remain only partially implemented in many institutions [13, 14, 15]. A recent publication from the GED taskforce, in collaboration with EDSA, helps to champion the concept and value of the ‘student voice’—and this is just one example of how our position has, quite rightly, changed over time [16]. These examples illustrate the growing recognition that curriculum development must be inclusive, flexible and responsive to evolving professional and societal realities.

In light of these changes, it is essential that the GED framework continues to be evaluated and refined to ensure it remains both relevant and fit for purpose. This paper describes the activities undertaken in 2024–2025 to convene a multi‐stakeholder dialogue on the future direction of Oral Health Professional [17] (OHP) education. Specifically, it reports on the methods and outcomes of a facilitated stakeholder event designed to gather diverse perspectives, identify implementation challenges and co‐develop shared priorities for the ongoing development of the GED framework.

## 2 Methodology

### 2.1 Study Design and Objectives

In early 2024, the GED Taskforce initiated the planning of a two‐day, in‐person stakeholder workshop aimed at critically examining the ideological foundations underpinning the GED curriculum framework. The primary objectives of the event were to:

1. Elicit stakeholder feedback to refine the GED approach.
2. Identify challenges in implementing the GED framework and in training OHPs.
3. Develop a shared, multi‐stakeholder perspective on priority actions and potential solutions to these challenges.

### 2.2 Participant Recruitment and Pre‐Event Preparation

**FIGURE 2:**
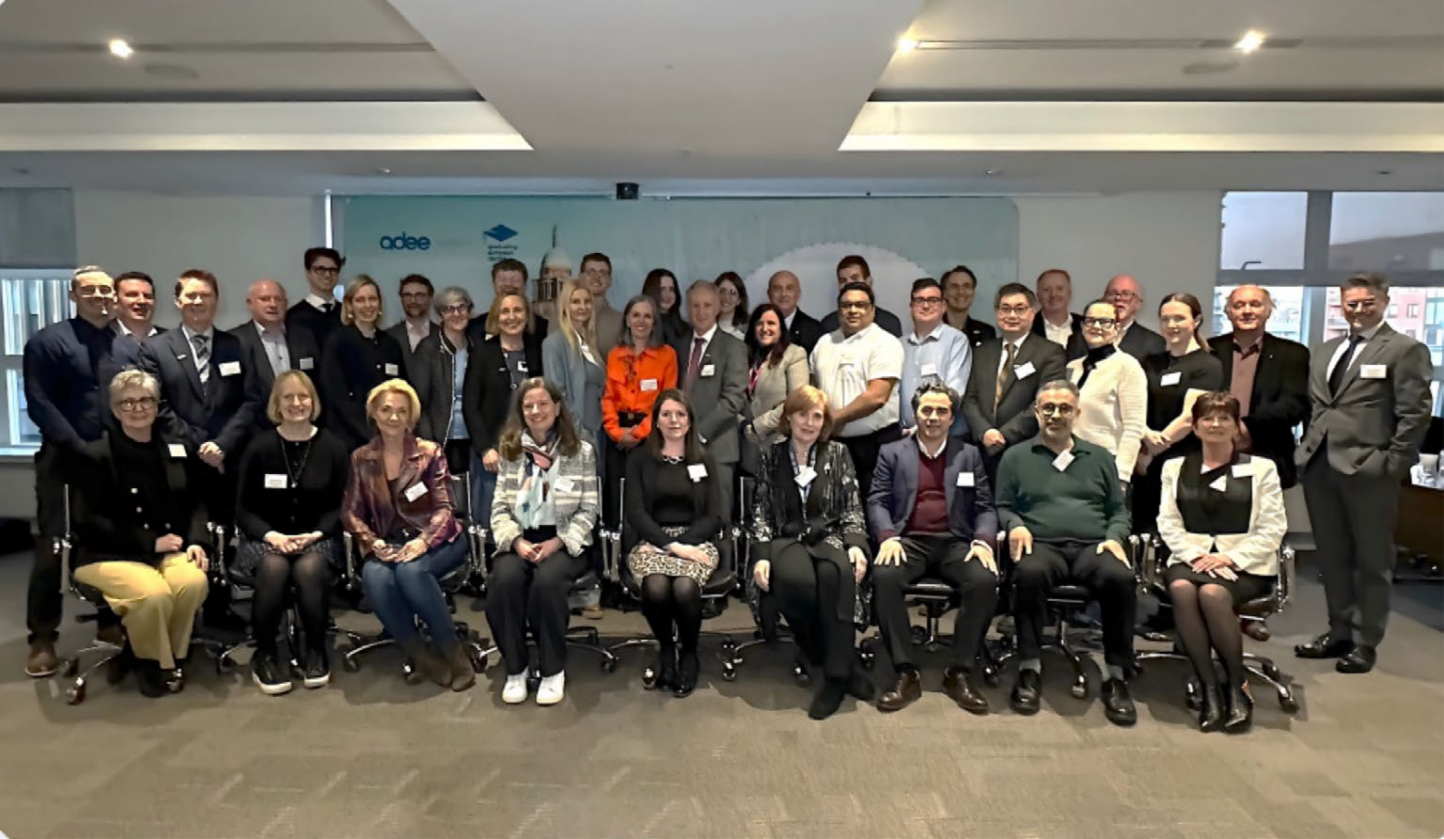
A selection of the delegate group.

The stakeholder event was held on 5th and 6th February 2025 in Dublin, Ireland. Invitations were distributed to a broad range of relevant stakeholders. A total of 38 stakeholders accepted the invitation (Appendix A), representing academic institutions, professional associations, regulatory bodies, public health organisations, students and industrial partners (Table 3, Figure 2). Participants' identities and institutional affiliations were documented, and informed participation was assumed through their voluntary registration and engagement.

**TABLE 3:** Organisations represented by the delegates.

Organisation type	Name
Educational organisations	ADEE Executive Committee
	ADEE GED taskforce
	International Federation of Dental Educators and Associations
	European Journal of Dental Education
	Association for Dental Education in America
Regulatory bodies and Government organisations	Council of the European Chief Dental Officers
	Department of Health, Ireland
	Federation of European Dental Competent Authorities and Regulators
	Council of European Dentists
	Dental Council of Ireland
	General Dental Council, UK
Student representative bodies	European Dental Students Association
Institutions	Royal College of Surgeons, Ireland
	Trinity College Dublin
	University College Cork, Ireland
	Szeged University, Hungary
	University of Liverpool, UK
	The University of Sheffield, UK
	Cardiff University, UK
	Malmo University, Sweden
	University Paris Cite, France
	KU Leuven University
	University of Jena, Germany
	University of Zagreb, Croatia
	The Arctic University of Norway
	ACTA, Netherlands
	University of Birmingham, UK
Specialty Boards, Societies and Organisations	Irish Dental Hygienist Association
	European Dental Hygiene Federation
	Federation Dentaire Internacionale (FDI)
	European Association for Dental Public Health
	Platform for Better Oral Health, Europe

In advance of the event, participants received preparatory materials, including selected readings, in order to provide theoretical grounding on curriculum ideologies:

- The Graduating European Dentist Curriculum Framework: A 7‐Year Review [5], https://onlinelibrary.wiley.com/doi/full/10.1111/eje.13058.
- Graduating European Dentist Curriculum Domain V: Research [11], https://onlinelibrary.wiley.com/doi/full/10.1111/eje.13040.
- The GED framework interactive online resource https://adee.org/graduating‐european‐dentist.
- O‐Health‐Edu: A vision for oral health professional education in Europe [18], https://onlinelibrary.wiley.com/doi/10.1111/eje.12819.

Participants were also invited to complete a short online survey designed to capture individual perspectives on key ideological orientations on OHP education. The results were used to inform thematic grouping of participants and to structure discussions during the workshop. The survey was based on a curriculum ideology framework adapted from Schiro's model [4], which identifies four principal orientations: Scholar Academic, Learner‐Centered, Social Efficiency and Social Reconstruction. To ensure relevance to the context of OHP education, the original ideology statements were modified to reflect learning in higher education settings specific to the OHP domain. Delegates were asked to rank statements under six thematic domains, according to their individual preferences (see Appendix B). This process enabled the taskforce to explore how different stakeholders prioritised educational values, and to align discussion accordingly during the Dublin 2025 event.

### 2.3 Event Facilitation and Format

To ensure impartiality and to foster open dialogue, the GED Taskforce appointed an independent facilitator (Ms Lisa Manselli) to moderate all sessions.

It was important to the Board and Taskforce that the core of the ideas, concepts, challenges and opportunities would come from the delegates rather than ADEE itself. For this reason, the underlying ethos was one that encouraged discussion, debate and agreed shared understanding. Mentimeter [19] was used to gather the stakeholders' perspectives.

## 3 Results

### 3.1 Stakeholder Preferences Regarding Curriculum Ideologies

Table 4 presents the distribution of delegates' first‐choice responses across the four curriculum ideologies adapted from Schiro's framework. The data indicate a clear inclination among participants towards a broad and diversified application of curriculum approaches. Notably, the Scholar Academic model was the least frequently selected as a primary orientation. This finding suggests a divergence between stakeholder perspectives and the traditional dominance of scholarly academic ideologies typically observed in higher education and professional training contexts. Instead, there was a marked preference for ideologies that emphasise the learner's experience, their societal utility and the role of education in addressing population health needs—namely, the Learner‐Centered, Social Efficiency and Social Reconstruction approaches. This shift highlights an emerging consensus that OHP education must evolve beyond content transmission towards socially responsive and student‐focused pedagogies.

**TABLE 4:** Suitable curriculum ideologies (Schiro) that were considered by the 2025 stakeholder delegates.

Curriculum element	Ideology, % respondents as first choice
Purpose	Socially efficient (44%) Socially reconstructive (40%) Learner centred (16%) Scholar academic—*no first choice*
Teaching	Learner centred (48%) Socially efficient (28%) Socially reconstructive (20%) Scholar academic (4%)
Learning	Learner centred (60%) Socially reconstructive (28%) Socially efficient (8%) Scholar academic (4%)
Content	Socially efficient (52%) Learner centred (20%) Socially reconstructive (16%) Scholar academic (12%)
Student outcomes	Socially reconstructive (40%) Socially efficient (36%) Scholar academic (20%) Learner centred (4%)
Evaluation	Socially efficient (48%) Learner centred (36%) Scholar academic (12%) Socially reconstructive (4%)

*Note:* The colours represent how popular each response was (green being the most popular, red being the least).

In summary, this provided a powerful basis for the ‘statement of the problem’ to conclude the first day; in that we need to work together as a group of stakeholders, to guide educators in shaping their programmes, their approaches and ultimately, their graduates' attributes.

### 3.2 Perceived Stakeholder Challenges in Delivering Quality OHP Education

**FIGURE 3:**
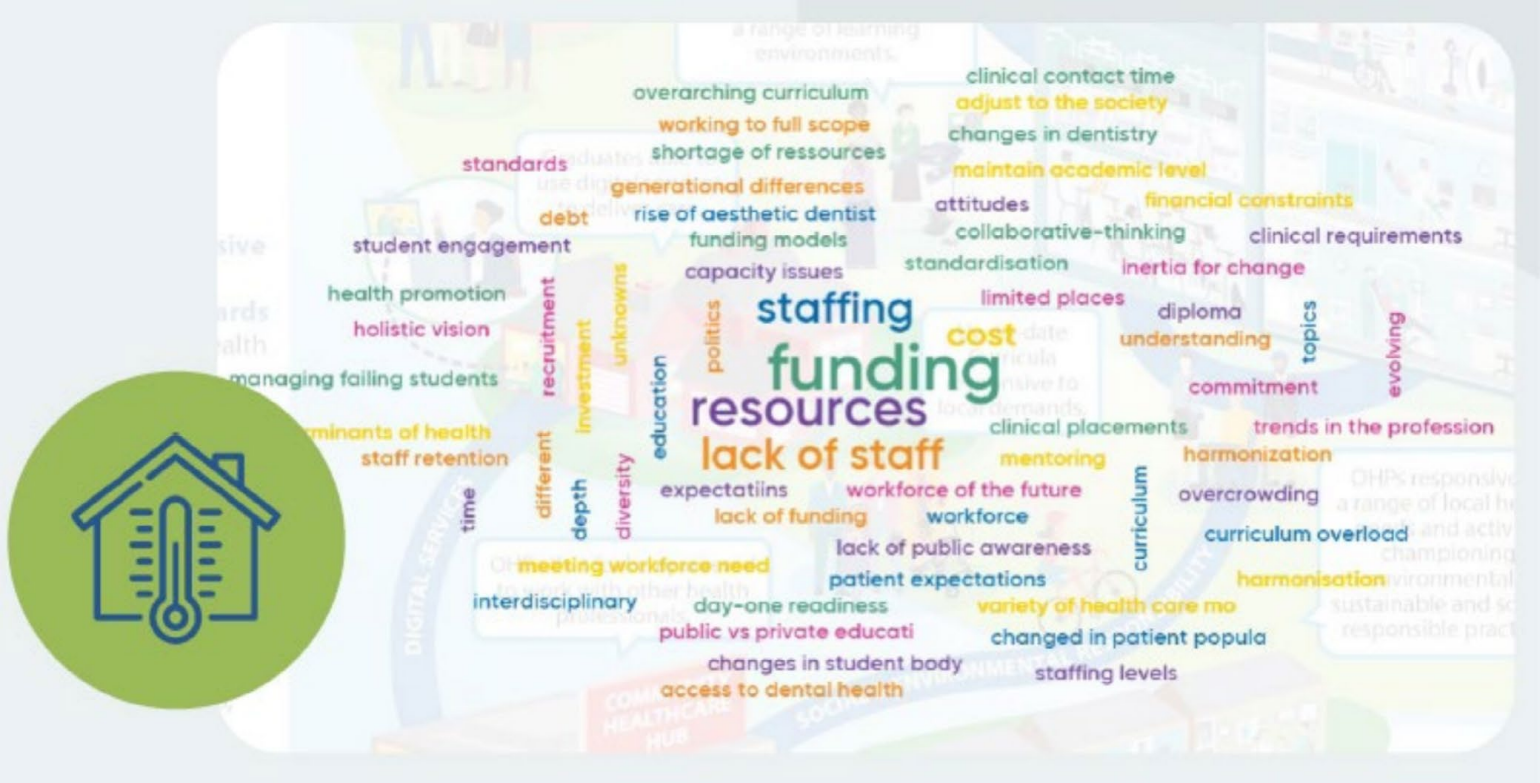
Word cloud from Mentimeter, showing the responses of delegates when asked about the challenges and opportunities in Oral Health Professionals' Education. Larger words indicate an increased frequency of use.

As part of the opening session, delegates were invited to participate in a live poll to share what they hoped to gain from the event. The most frequently cited expectations included establishing a shared understanding of priorities and fostering the exchange of knowledge and professional insights. A second poll focused on identifying the current challenges faced in delivering high‐quality OHP education. Delegates were asked to respond based on their own institutional and professional experiences, informed by the preparatory reading materials, including the GED framework and associated documents. The responses revealed a wide range of perceived barriers, with particular emphasis on funding limitations, resource constraints and staffing shortages. Nonetheless, the breadth of issues highlighted by participants extended beyond structural concerns to include student‐related challenges such as educational debt, academic underperformance and the management of struggling learners. A visual summary of these responses is illustrated in Figure 3, which presents a word cloud generated from the submitted data.

Following further discussion, 3 themes, with associated challenges, were presented for exploration at the meeting:

1. Student experience and patient safety
  - Limitations with patient mix/amount/level of clinical experience
  - Limits on clinical contact time
  - Limited staff numbers/poor ratios/lack of expertise
2. Social efficiency and the workforce
  - Ensuring appropriate recruitment and admission of students
  - Ensuring the health needs of the population are met
  - Delivering true Inter‐Professional Education
  - Preparing students to work in a particular Healthcare system
  - Lack of enthusiasm for working in a state sector
  - Catering for changing workforce requirements
3. Curriculum approaches
  - Disparate curriculum approaches across the European region
  - Lack of student independence upon graduation
  - Early identification of struggling students
  - Increases in student requests for support
  - Managing students who are failing to progress

### 3.3 Perceived Priorities and Impacts

**FIGURE 4:**
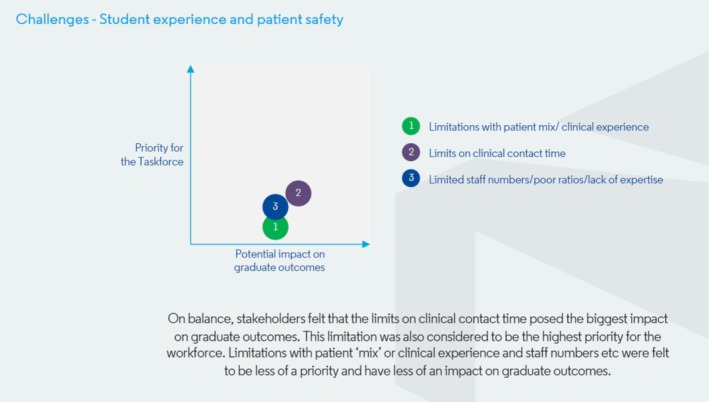
The way in which delegates ranked the potential impacts of challenges related to student experience and patient safety, and to what extent they saw them as priorities for the taskforce.

**FIGURE 5:**
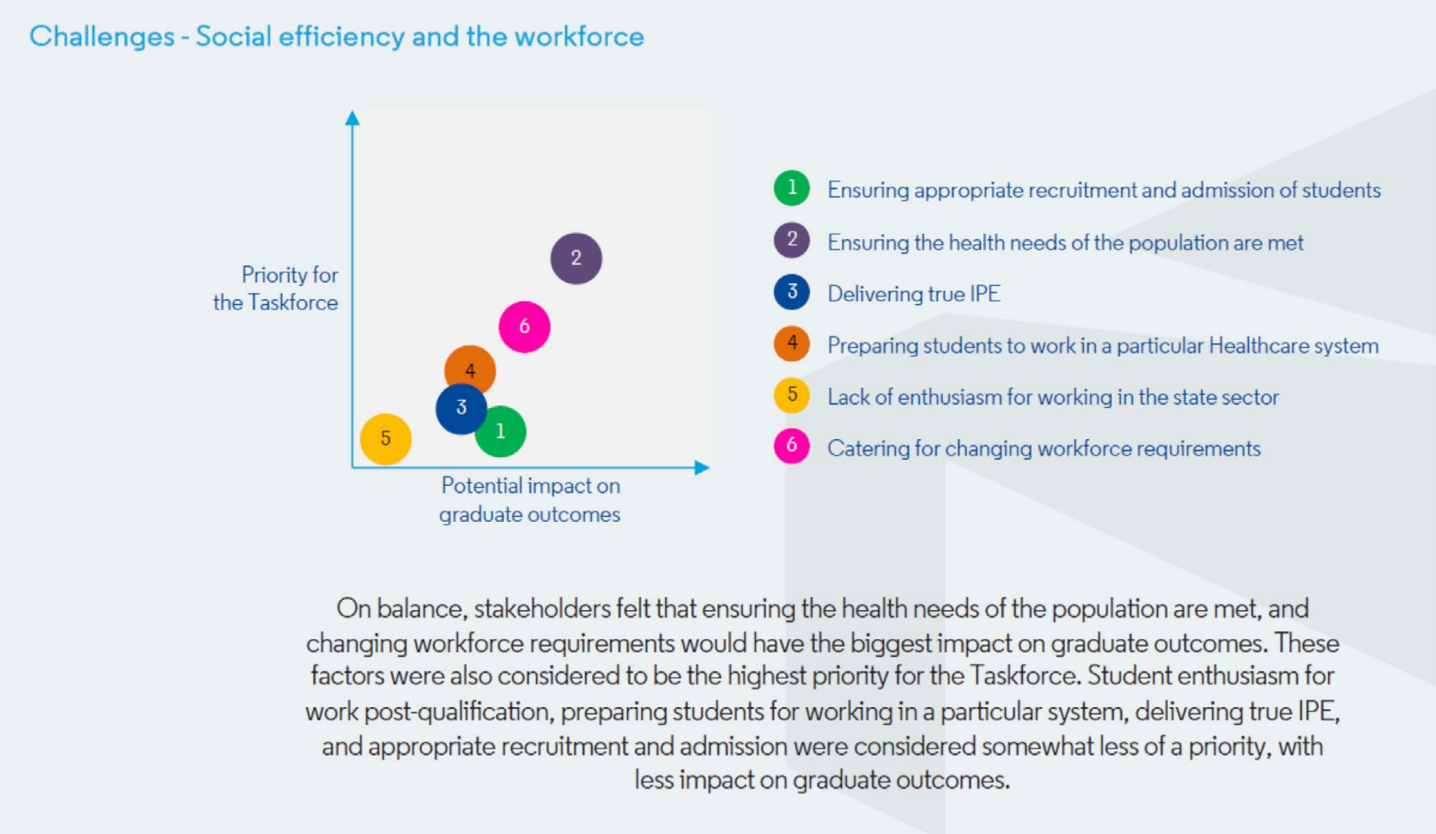
The way in which delegates ranked the potential impacts of challenges related to social efficiency and the workforce, and to what extent they saw them as priorities for the taskforce.

**FIGURE 6:**
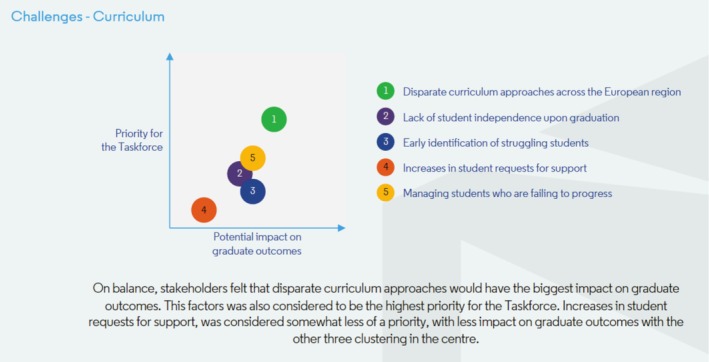
The way in which delegates ranked the potential impacts of challenges related to the curriculum, and to what extent they saw them as priorities for the taskforce.

Delegates discussed the potential impact that the challenges could have on graduate outcomes—and the extent to which the challenges were seen as priorities for the taskforce. The way in which delegates ranked the potential impacts of each challenge, and to what extent they saw them as priorities for the taskforce, is represented in Figures 4, 5, 6. The taskforce was mindful that presenting the data in this way represented an average view of stakeholders—and that individual stakeholder views may differ significantly. As such, these findings were followed up with extensive group discussion on Day 2.

## 4 Day 2

On Day 2, the delegates considered the higher‐priority challenges identified on Day 1 (below), working in groups to derive potential solutions to each challenge.

Theme One: *Challenges with student experience and patient safety*.

Theme Two: *Challenges with meeting the health needs of the population*.

Theme Three: *Challenges with variability of curriculum approaches across Europe*.

Theme Four: *Challenges with early identification and managing students who are failing to progress*.

Theme Five: *Challenges with lack of student independence on graduation/preparing for practice*.

Theme Six: *Challenges with a changing workforce*.

Theme Seven: *Challenges with student recruitment and admissions*.

Each group's work was facilitated by a member of the Taskforce, to enable them to capture and carry forward their views. This ensured that discussions remained as true as possible to each stakeholder's view—supporting an ethos of collaboration and shared understanding. Individuals then reported their levels of support for each potential solution, using Mentimeter. The potential solutions for each challenge and their levels of support are represented in Table 5.

**TABLE 5:** Potential solutions for each challenge, and their general levels of support (highest support at the top, light green; very appropriate, dark green; appropriate, amber; neutral, red; not appropriate).

Theme	Solution	Support
Student experience and patient safety	Increases in practical simulation (skills lab)	
	Increases in virtual reality simulation	
	Increases in case‐based discussions	
	Focus on areas of capability instead of specific numbers of procedures	
	Increases in outreach placements	
	Student exchanges to centres with more practical opportunities	
	Earlier commencement of practical clinical skills	
	Increased use of shared academic resources for teaching	
	Patient incentives to come for treatment	
	More strict use of student time	
	Increase salaries to recruit staff	
	Reduction in clinical requirements	
	Increase in programme length/duration	
	Lowering recruitment standards for staff	
	Additional teaching for staff & reduction in scholarship/research time	
	Reduction in clinical contact time for students	
Meeting the health needs of the population	Increase the number of outreach centres in areas of treatment need	
	Community initiatives to raise awareness regarding the oral health context locally	
	Curriculum focus on Interprofessional Education	
	Training students for remote access to healthcare/digital dentistry	
	External placements to other allied health professions	
	Curriculum focus on WHO and other health strategies	
	Reduce student entry requirements in areas of high treatment need	
	Patient and public participation in development of the curriculum	
Variability of curriculum approaches across Europe	Development of an expected standard of clinical training	
	Develop guidelines on longitudinal clinical evaluation	
	Alignment to a common approach (i.e., GED)	
	Alignemnt of national regulatory approaches	
	Amend the EU directive for greater clarity on learning outcomes	
Early identification, and managing students who are failing to progress	Longitudinal monitoring of behaviours and appropriate interventions/management pathways	
	Early practical skills development	
	Earlier clinical contact	
	Increased contact time and monitoring with academic staff	
	Strict requirement to pass gateway assessments early in the programme	
	Early exit awards for failing students	
	Supporting repeatedly failing students to repeat their studies	
Lack of student independence on graduation/preparing for practice	Early clinical exposure	
	Longitudinal curriculum focus on reflective practice	
	Post‐qualification training/mentorship	
	Additional year of study/6 year programme	
	More focus on co‐creation of programmes with students	
	Tiered health care systems/limiting scope of practice	
Challenges with a changing workforce	Introduction of ‘mid‐level’ professionals	
	Educate more dentists	
	Commitment to health service post‐graduation	
	Reduction in training of dentists and increased training of mid‐level professionals	
	Reduction in training of dentists and increased training of dental hygienists	
	Reduction in training of dentists and increased training of medical professionals in oral health care	
Challenges with student recruitment and admissions	Increased range of selection processes	
	Graduate entry programmes	
	Ask for prior experiences (e.g., working as an assistant)	
	Increase in entry requirements	
	Reduction in entry requirements	

### 4.1 Key Initiatives

In assimilating these considerable and varied initiatives, the taskforce clearly has a multiparty mandate to ensure that the GED continues to be of value and use to its stakeholders. Given the diversity not only of the stakeholder requirements and expectations, but also of regional and national variation in the delivery of OHP education throughout Europe, ensuring regional participation in the Taskforce's work and enabling an inclusive approach to updating and the development of future supporting resources will be key. Whilst all proposed suggestions were valid, the taskforce has considered the full range of discussions across the stakeholder event. The Taskforce has prioritised a number of initiatives that it believes will help to address significant challenges in the delivery of OHP education in coming years.

With this in mind, the Taskforce proposed the following objectives for 2025–2030:

- Expand taskforce membership to drive regional representation in future work.
- Establish subgroups reporting to the Taskforce on the development of guidance on GED use by regulators and institutions.
- Establish a subgroup to explore the development of an expected standard for clinical training and contact time.
- Establish a subgroup to explore the suitability and practicality of the development of a common curriculum for OHP education and how this might align within the GED.
- Consider how aspects such as reflective practice, outreach, digital dentistry, AI and the other recurring discussion themes can be best integrated within the GED and existing resources.
- Continue the evolution of ADEE MOLAR—the curriculum mapping platform.
- Continue to actively engage with pan‐European and regional key partners on the GED's evolution, and to enable greater awareness of the project.

As the meeting drew to a close, the taskforce summarised the findings and outlined the next steps.

## 5 Summary

In 2025 the GED taskforce held a multi‐stakeholder event that undertook a deep dive into the perceived ideologies underpinning OHP education. This paper reports how the event was planned and conducted—and reports the challenges that were discussed in relation to delivering OHP education, potential solutions to each challenge and priorities for which the taskforce should focus its activity. Due to the very collaborative and fruitful nature of this event, ADEE plans to hold further multi‐stakeholder meetings across Europe.

## Conflicts of Interest

The authors declare no conflicts of interest.

## Data Availability

Research data are not shared.

## Appendix A Attendee List

Barry Quinn ADEE Board Secretary General & The University of Liverpool, United Kingdom.

Brian O'Connell ADEE President & Faculty of Health Sciences Trinity College Dublin, Ireland.

Corrado Paganelli IFDEA Board & The Council of European Chief Dental Officers, Italy.

Mª Cristina Manzanares Editor EJDE & University of Barcelona, Spain.

Ina Schüler ADEE Board & University of Jena, Germany.

Ivan Alajbeg ADEE Board President Elect & University of Zagreb, Croatia.

James Field ADEE Board & Cardiff University, United Kingdom.

Jonathan Dixon GED Taskforce & The University of Sheffield, United Kingdom.

Julia Davies ADEE Board Treasurer & Malmo University, Sweden.

Katleen Van Damme ADEE Board & KU Leuven, Belgium.

Mohammad Al Horani ADEE Board & UiT The Arctic University of Norway, Norway.

Ronald Gorter ADEE Board & ACTA Amsterdam, Netherlands.

Sibylle Vital ADEE Board & University Paris Cite, France.

Upen Patel ADEE Board & University of Birmingham, United Kingdom.

Albert Leung RCSI Dental School, Ireland.

Barry Crossan Department of Health, Ireland.

Blanaid Daly Dublin Dental University Hospital, Trinity College Dublin, Ireland.

Cedric Grolleau FEDCAR, France.

Clara Luciani CED Secretariat, Belgium.

Derek Sullivan, Trinity College Dublin, Ireland.

Dympna Kavanagh Department of Health Ireland, Ireland.

Emma Ryan Irish Dental Hygiene Association, Ireland.

Filip Galo EDSA Board, Slovakia.

Gitana Rederiene EDHF Board, Lithuania.

Gráinne Ginty Accreditation Manager, Irish Dental Council.

Jack Nagle Alpha Consulting (Department of Health of Ireland), Ireland.

Katalin Nagy CED Board & Szeged University, Hungary.

Maria João Ponces FEDCAR Board, Portugal.

Marsha Pyle ADEA representation, United States.

Max Walsh EDSA Irish Rep, Ireland.

Michael Dolan Department of Health, Ireland.

Miguel Pavão FEDCAR Board, Portugal.

Paul Brady University College Cork, Ireland.

Paul Lyons Irish Dental Council, Ireland.

Ross Scales General Dental Council UK, United Kingdom.

Saulė Skinkytė EDSA Board, Lithuania.

Simona Dianiskova FDI‐ERO, Slovakia.

Stephanie Tubert Jeannin EADPH Board, France.

## Appendix B Modified Curriculum Ideology Statements

**Table:**

Curriculum element	Ideology (hidden)
*Teaching*	
Teachers should be facilitators for students learning, helping them by presenting them with real life experiences from which they can make meaning	Learner Centred
Teachers should be knowledgeable people, transmitting that which is known, to those that do not know it	Scholar Academic
Teachers should be supervisors of student learning and student patient care, and use strategies that optimise student learning	Social Efficiency
Teachers should see students as junior colleagues, using the environment within which they operate to help students learn	Social Reconstruction
*Learning*	
Learning best takes place when students are motivated to actively engage in experiences that provide context to their learning	Learner Centred
Learning best proceeds when the teacher clearly and accurately presents the knowledge that is to be acquired	Scholar Academic
Learning best proceeds when the student is presented with the appropriate stimulus materials and positive reinforcement	Social Efficiency
Learning best occurs when a student confronts a real world crisis and participates in the construction of a solution	Social Reconstruction
*Content*	
The knowledge of most worth is that which comes from direct experience, and personal responses to that experience	Learner Centred
The knowledge of most worth is the structured knowledge and ways of thinking that have come to be valued over time	Scholar Academic
The knowledge of most worth is the specific skills and capabilities that allow an individual to enact a constructive professional life	Social Efficiency
The knowledge of most worth is a set of social ideals, a commitment to those ideals, and an understanding of how to implement them	Social Reconstruction
*Student outcomes*	
The curriculum should facilitate students unfolding as learners according to their own innate, felt needs. The focus is on students as they are during their studies, not as they might be as dentists	Learner Centred
The curriculum should facilitate intellectual development highlighted by growing reasoning ability and capacity for memory	Scholar Academic
The curriculum should prepare students for becoming a dentist, when one will be a constructive, contributing member of society	Social Efficiency
The curriculum should facilitate practice and preparation for acting upon the needs of society to improve the students professional selves, and the health of society	Social Reconstruction
*Evaluation*	
Evaluation should continuously assess student needs	Learner Centred
Evaluation should objectively determine the amount of knowledge students have acquired	Scholar Academic
Evaluation should objectively indicate to others whether or not students can or cannot perform specific skills	Social Efficiency
Evaluation should be a subjective comparison of student performance with their capabilities, and a judgement about how they are ‘living up’ to their capabilities	Social Reconstruction
*Purpose*	
A school should be enjoyable, stimulating, student‐centred and organised around the development needs of the student themselves	Learner Centred
A school should be a community where the accumulated knowledge of Dentistry is transmitted to our students	Scholar Academic
A school should fulfil the oral health care needs of society by efficiently training our students to function as constructive members of the oral health work force, in society	Social Efficiency
A school should provide students with the ability to perceive problems with oral health care in society, have a vision for a better functioning society, and act to change society so that there is better oral health, and a better life for all people	Social Reconstruction
